# General practitioners' experience and benefits from patient evaluations

**DOI:** 10.1186/1471-2296-12-116

**Published:** 2011-10-31

**Authors:** Hanne N Heje, Peter Vedsted, Frede Olesen

**Affiliations:** 1 The Research Unit and Department of General Practice, University of Aarhus, Aarhus, Denmark

## Abstract

**Background:**

It has now for many years been recognised that patient evaluations should be undertaken as an integral part of the complex task of improving the quality of general practice care. Yet little is known about the general practitioners' (GPs') benefit from patient evaluations. Aim 1 was to study the impact on the GPs of a patient evaluation and subsequent feedback of results presented at a plenary session comprising a study guide for the results and group discussions. Aim 2 was to study possible facilitators and barriers to the implementations of the results raised by the patient evaluation process.

**Methods:**

A patient evaluation survey of 597 voluntarily participating GPs was performed by means of the EUROPEP questionnaire. Evaluation results were fed back to the GPs as written reports at a single feedback meeting with group discussions of the results. Between 3 and 17 months after the feedback, the 597 GPs received a questionnaire with items addressing their experience with and perceived benefit from the evaluations.

**Results:**

79.4% of the GPs responded. 33% of the responding GPs reported that the patient evaluation had raised their attention to the patient perspective on the quality of general practice care. Job satisfaction had improved among 26%, and 21% had developed a more positive attitude to patient evaluations. 77% of the GPs reported having learnt from the evaluation. 54% had made changes to improve practice, 82% would recommend a patient evaluation to a colleague and 75% would do another patient evaluation if invited. 14% of the GPs had become less positive towards patient evaluations, and job satisfaction had decreased among 3%.

**Conclusions:**

We found a significant impact on the GPs regarding satisfaction with the process and attitude towards patient evaluations, GPs' attention to the patients' perspective on care quality and their job satisfaction. Being benchmarked against the average seemed to raise barriers to the concept of patient evaluations and difficulties interpreting the results may have formed a barrier to their implementation which was partly overcome by adding qualitative data to the quantitative results. The GPs' significant willingness to share and discuss the results with others may have served as a facilitator.

## Background

Patient evaluations have become an integral part of the quality assessment of health care. By basing the methods for patient evaluations on studies of patients' priorities regarding the quality and by singling out aspects of care that are particularly important from their perspective, patients become a crucial source of information in quality improvement efforts [1,2]. In order for the assessment results to serve as an instrument for improving care the results must be fed back to the care providers in a manner that eases their transposition into concrete initiatives to support quality improvement efforts in practice. Improvement of general practice care based on such assessment requires that the general practitioner (GP) is motivated for change and is sensitive to patients' opinions. The evaluation process therefore should not raise barriers for using the results, and the feedback should be immediately interpretable by the evaluated GPs [3,4].

The literature in the field of feedback of results from patient evaluations is sparse. Two studies from hospital settings suggest that frequent feedback of survey results followed by regular team discussions alone may lead to improvements in the patients' experience of care [5,6]. Tasa et al. carried out focus group interviews with representatives from a hospital setting who had been using patient evaluations and feedback studying barriers towards the implementation of patient assessment results. From this study it was suggested to provide both quantitative and qualitative data and to present feedback in a comprehensible format that allowed identification of care aspects in need of improvement. The study also suggested an organisational strategy for the feedback and implementation of results [7]. In their qualitative interview study and literature review from 2005 also Davies and Cleary concluded that implementation of patient survey results in a hospital setting required a supportive organisation [8].

In 2007 Evans et al published a review of patient survey instruments designed for general practice and found that results from the applications of six of these validated instruments had been used to produce individual written feedback reports to the evaluated GPs [9]. The impact of feeding back results was studied for two of them: the CEP [10] and the IPQ [11]. Greco et al. found no measurable effect of patient feedback on the interpersonal skills of GPs [12] but a positive effect on that of GP registrars [13] when using the IPQ. In a Dutch patient evaluation study using the CEP with written feedback of individual and aggregated results together with non-individualised suggestions for improvements, the GPs found the results difficult to use and the feedback had no effect on the subsequent patient evaluations. Only 24% of evaluated GPs found patient evaluation surveys useful and it was suggested to combine feedback with small group education [14,15]. Among the survey instruments for which the impact of feeding back results to the care providers has not been studied is the widely used EUROPEP instrument [16,17].

Remembering the results of the study by Tasa et al. [7] it seems reasonable to suggest that providing written feedback of assessment results to the evaluated GPs may not be sufficient to produce improvements. Wensing et al. [14] suggest a combination with small group education but there may be other supportive initiatives to facilitate the GPs' implementation of their evaluation results.

On this background the present study had two aims: One was to study the impact on the GPs of a patient evaluation and subsequent feedback of results presented at a plenary session comprising a study guide for the results and group discussions assuming that an experienced benefit from a patient evaluation is the first step towards practice improvement (aim 1). The other was to study possible facilitators and barriers to the implementations of the results raised by the patient evaluation process (aim 2).

## Methods

The present study was based on a national patient evaluation project with voluntary participation using an internationally validated questionnaire to measure the patient-experienced quality of general practice care. Written feedback was presented to and discussed with the GPs. The organisation of Danish general practice is outlined in Additional file 1.

### Study population

In 2002-4, all 2361 GPs in eleven Danish counties were - county by county - invited to carry out patient evaluations of their practices through participation in a Danish nationwide patient evaluation project, the DanPEP. The invitation was accompanied by written information about the project and an invitation to participate in an informational meeting. A total of 597 GPs (between 16 and 66% of all GPs in the participating counties) entered the study. According to recommendations from earlier studies, we encouraged the GPs to meet with groups of colleagues to follow up on the results of the evaluation [14]. In each county, a GP was selected by the local committee for quality improvement to act as contact person between the GPs and the project secretariat.

The participating GPs from ten counties (n = 482) handed out questionnaires to 100 successive adult patients from the GP's own list seen by the GP in the clinic or at home visits. Reminders were mailed to non-responding patients. The response rate was 83,4%. Due to a concomitant study of the effect of reminders [18] 117 of the GPs handed out 130 questionnaires, but did not use reminders. Using this method we attained a response rate of 65,7%. Their evaluations were therefore based on approximately the same number of responses as those in the first group. In the last county we carried out another nested study comparing results from a postal survey with the results from the handed out survey, which was why 115 GPs were evaluated through a postal survey 150 patients per GP drawn by the regional health registry from lists of patients who had been consulting the participating GPs. Each questionnaire was - disregarding the method of distribution - identified by a serial number connecting it with the relevant participating GP. In this part of the study the response rate was 64,6%.

### Patient evaluation

The questionnaire contained the 23 items forming the EUROPEP instrument [16,17] which addresses aspects of the doctor-patient relationship, medical care, information and support, organisation of care and accessibility. Three open-ended questions concluded the questionnaire: "What do you think is good about your GP?", "What do you think your GP could change or do better?" and "Do you have any other comments?" The study design allowed the patients' anonymous assessment of individual GPs.

### Feedback

The GPs received a formal report with their individual evaluation results presented as the percentage of answers in each category. For comparison they also received the average group results, if the GP was a member of a group, and the county average results presented in the same way. In order to allow identification of aspects of care in need of improvement [7], we presented the results at the single item level. The results were not adjusted for the heterogeneity of patient lists or for GP and practice characteristics. For 267 GPs, the feedback also included the patients' written answers to the above-mentioned open-ended questions. In each county all the evaluated GPs were invited to a meeting where the feedback was presented. In counties with many participating GPs we held up to three meetings thus enabling a more intimate environment (still, each GP only attended one meeting). In the first part of each meeting the GPs were guided through the interpretation of the tables and figures contained in the reports. In the second part of each meeting we conducted so-called cafe-sessions with different combinations of GPs thus enabling each GP to share his or her reflections with as many colleagues as possible regarding topics from the evaluations chosen by the project group. By doing so we intended to encourage the GPs' reflection on how to transpose the results into quality improving activities in their daily practice.

### The GPs' evaluation of the project

Between 3 and 17 months after the feedback, the GPs received a questionnaire addressing their experience with and perceived benefit from the patient evaluation. The questionnaire included items evaluating the process thus addressing the GPs' attitude towards patient evaluations, their considerations prior to the evaluation, and their experience with the evaluation process and with the feedback. The questionnaire also included items evaluating the outcomes addressing the GPs' activities following the patient evaluation and their experienced benefit from the project. Each questionnaire was identified by a serial number linking it to the result of the GP's evaluation.

### Analyses

Differences between groups of GPs were tested using the Chi square test. Whenever analyses included patient evaluation results, we used an average score expressed as a percentage of the maximum possible score and dichotomised according to average.

### Ethics

This project fell outside the scope of the Danish rules for ethical approval.

## Results

A total of 597 questionnaires were distributed to the GPs and 474 (79.4%) valid responses were returned. Among the 267 GPs whose feedback included the patients' replies to the open-ended questions, 212 (79.4%) GPs responded.

### Information

Half of the GPs attended the local informational meeting and more than three quarters felt well-informed about the project (Table 1). A statistically significant majority of those who did not feel well-informed had not attended the meeting (64.7% vs. 35.3%, p = 0.012).

**Table 1:** GPs' assessment of information provided prior to their patient evaluation (n = 474)

	**Yes**	**No**	**Missing**
	
Did you attend the informational meeting prior to the patient evaluation in your region?	47.9	47.5	4.6
	Agree or mostly agree	Disagree or mostly disagree	Missing
	
I felt well-informed about the project prior to my patient evaluation	83.3	10.8	2.1

(% of answers)

### Groups

More than half of the GPs attended the study as part of a group (Table 2) and three quarters of these GPs found group attendance beneficial. 38% of the GPs who did not participate as a group thought that they might have benefited from attending a group.

**Table 2:** GPs' assessment of group participation (n = 258) or non-participation (n = 210)

GPs attending a group (% of answers)			
	Yes	No	Missing
	
Did you benefit from attending a group?	71.3	24.4	5.0
Would you attend a group again if you were given the opportunity to choose?	88.0	6.2	5.8
**GPs not attending a group** (% of answers)			

	Agree or mostly agree	Disagree or mostly disagree	Missing
	
I think I may have benefited more from my patient evaluation if I had attended a group	38.1	47.1	14.8
I would attend a group if I were given the opportunity to choose	34.3	50.0	15.7

### Feedback

Nearly three quarters of the evaluated GPs attended the feedback meeting (Table 3). More than half of the evaluated GPs found their results difficult to interpret in relation to everyday practice, irrespective of whether they had attended the feedback meeting or not. GPs who also received their patients' replies to the open-ended questions had less difficulty interpreting the results (37.8% vs. 50.2%, p = 0.007). Statistically significantly more GPs who found it unpleasant to receive the evaluation results had attended the feedback meeting (83.6% vs. 16.4%, p = 0.013) and had evaluation results below average (74.6% vs. 25.4%, p < 0.001) compared with GPs who did not perceive the experience as unpleasant. A minority did not discuss the results with anyone.

**Table 3:** GPs' assessment of feedback and how they handled it (n = 474)

The feedback (% of answers)			
	Yes	No	Missing
	
Did you attend the feedback-meeting following the patient evaluation in your region?	70.7	29.1	0.2
	Agree or mostly agree	Disagree or mostly disagree	Missing
	
It was unpleasant for me to receive the results of my patient evaluation	14.1	84.4	1.5
It was difficult to interpret the results of my patient evaluation and to apply the results in my practice	55.3	42.4	2.5
**With whom did you discuss the results of your patient evaluation?** **To whom did you show your feedback-report?** (% of answers, the GPs were allowed to place more than one x)			

	I discussed the results with	I showed my report to	
	
The group of colleagues with whom I participated in the project	55.5	43.0	
The colleagues in my practice	71.9	63.7	
The staff in practice	62.7	48.3	
My spouse or others in my family	54.2	40.5	
One or more of my patients	7.8	1.7	
Others	12.0	6.5	
No one	1.3	9.5	

### Benefit

Three quarters of the GPs reported having learnt something from the evaluation that they could apply in their practice (Table 4). All in all, 60.5% had either already carried out or specifically planned changes or other activities, while one third had not done or planned anything and did not consider to. Changes or other activities were most often related to aspects of accessibility, which was the dimension that received the poorest assessment in general. Examples of changes and plans are displayed in Table 5. All changes made by the GPs are displayed in the Additional file 2 "Changes in practice". One fifth of the GPs had made changes and plans regarding interpersonal aspects of care. The propensity to make changes and plans was not associated with the degree to which the GPs felt well-informed prior to the evaluation, with their attending a group or with the way that they had experienced the feedback process. However, the propensity to make changes rose if the evaluation was below average (67.3% vs. 57.8%, p = 0.032), if the GPs had received the patients' replies to the open-ended questions (68.4% vs. 57.0%, p = 0.010) and if they had attended a feedback meeting (66.0% vs. 48.6%, p < 0.001).

**Table 4:** GPs' reported benefit from patient evaluation and actions taken (n = 474)

Changes, activities and plans owing to the patient evaluation (% of answers)				
	Yes	No		Do not know or missing
	
Have you learned anything from your patient evaluation that you can apply to your practice?	77.0	11.6		11.4
	Yes	No		Missing
	
Have you made changes or other activities in your practice due to your patient evaluation?	54.2	45.1		0.6
Have you planned specific changes or other activities in your practice due to your patient evaluation?	25.3	73.2		1.5
Have you either made changes or other activities in your practice or made specific plans to do so or both?	60.5	39.0		0.4
Are you still considering what to do due to your patient evaluation?	25.1	73.0		1.9
**Under which heading would you categorise your changes, activities and plans?** (% of answers, the GPs were allowed to place more than one x)				

	Changes and activities		Specific plans	
	
Doctor-patient relation	21.1		4.2	
Medical care	5.1		4.0	
Information and support	12.9		5.5	
Organisation of care	15.8		7.6	
Accessibility	36.5		20.0	
Other	4.4		2.3	
**How did you benefit from your patient evaluation compared with your expectations?** (% of answers)				

	As or more than expected		None or less than expected	Missing
	
Professionally	48.5		48.9	2.5
Regarding the quality of the doctor-patient relation	71.1		25.7	3.2
Regarding practice service towards the patients	76.8		20.5	2.7
Personally	70.7		26.4	3.0
Relation between colleagues	56.3		40.3	3.4
**Awareness of patient perspective** (% of answers)				

	Agree or mostly agree		Disagree or mostly disagree	Missing
	
The patient evaluation has increased our awareness in practice that the patients' perspective on quality in general practice may differ from ours	33.3		54.9	11.8
The patient evaluation has increased the awareness of GPs and practice staff of the quality of our work	32.9		57.0	10.1
The patient evaluation has increased our readiness for changes in practice	40.1		45.1	14.8
**Job satisfaction** (% of answers)				

	Better	Unchanged	Worse	Missing
	
Due to my patient evaluation my job satisfaction has become	26.4	69.0	3.2	1.5

**Table 5:** Examples of changes and activities caused by the evaluation*

Doctor-patient relation	"I try to listen to the patient in a more open-minded manner" "I try to appear less busy than I feel" "I try to seem more present minded during the consultation" "I try to conduct a more direct and authoritative consultation style" "I passed a one year psychotherapeutic training course"
Medical care	"We plan to do more quality assurance of our procedures" "We have extended the duration of a standard appointment" "I intend to be more focused on my role as the medical expert during the consultation"
Information and support	"I have become more thorough in my information to the patient about examinations and treatment" "I have become more aware of the importance that the patient understand and accept a plan for examination and treatment" "We produced written information and instruction to the patients"
Organisation of care	"We doctors confer more about difficult and shared patients" "We have introduced staff meetings where we discuss our routines and patient cases" "We are in a process of allocating duties and rearranging working hours" "We introduced a more flexible schedule"
Accessibility	"We introduced the possibility to book an appointment, renew a prescription or have an e-mail-consultation via our homepage"** "We allocated an hour a day for consultations without appointment" "We made more appointments possible in our schedule" "We extended the time for telephone consultations" "We tried to be more disciplined when speaking with patients in the telephone in order to solve matters quicker" "We have become better at keeping to the schedule" "We have more incoming telephone lines and more staff to answer the phone"
Other	"We redecorated the waiting room" "We moved to larger premises" "We discussed our results with the colleagues in our CME-group and in our supervision groups"

* A complete tabulation of all the changes made by the GPs following their patient evaluation is displayed in "Additional file 2: Changes in practice"

**These are services for which the GP-contract offers a fee

More than half of the GPs had benefited as expected or more than expected, mostly in relation to practice service towards the patients and the doctor-patient relationship (Table 4). Around one third of the GPs found that the patient evaluation had changed their view on the patient perspective on quality in practice, irrespective of whether they had received the replies to the open-ended questions or not. Around one quarter of the GPs reported that their job satisfaction had increased, but 3% found that their job satisfaction had declined. An increase in job satisfaction was associated with an evaluation result above average (30.8% vs. 21.7%, p = 0.029) and with not attending a group (22.9% vs. 31.1%, p = 0.048). A decrease in job satisfaction was associated with an evaluation result below average (5.8% vs. 1.2%, p = 0.005), but not with group attendance.

### Present attitude

One fifth of the GPs had adopted a more positive attitude towards patient evaluations during the process, whereas 14.0% had become less positive (Table 6). 82% would or would probably recommend a patient evaluation to a colleague and three quarters would or would probably repeat the evaluation if invited. An evaluation result above average was associated with a more positive attitude towards patient evaluations (25.3% vs. 16.7%, p = 0.023) and with a propensity to recommend an evaluation (91.1% vs. 84.1%, p = 0.024), but not with the willingness to repeat the evaluation.

**Table 6:** GPs' reported attitude to patient evaluations after being evaluated (n = 474)

	**More positive**	**Unchanged**	**Less positive**
	
My present attitude towards patient evaluations compared with my attitude prior to the evaluation	21.4	64.3	14.0
	Yes	No	I do not know
	
Would you recommend a patient evaluation to a colleague?	82.3	11.2	6.5
Would you do another patient evaluation in 3 years if you were invited to do so?	75.4	18.8	5.5

(% of answers)

## Discussion

We found a significant impact on the GPs of undergoing a patient evaluation with subsequent feedback as it was set up in the DanPEP study (voluntary informed participation, validated questionnaire, individual written feedback with quantitative as well as qualitative data, a single feedback meeting with group discussions of the results), that is, a majority reported specific benefit regarding certain aspects of practice and that they had learnt from their evaluation. A majority had made changes in practice or had specific plans to do so, and a significant fraction had experienced an increased awareness to the patient perspective on services. The immediate satisfaction with the process was contained in the responses to the questions "Would you recommend a patient evaluation to a colleague?" and "Would you go through another patient evaluation in three years if you had the opportunity?" to which questions 82% and 75% respectively asked "Yes" or "Most likely". Hence the majority had a positive experience. As participation in the project was voluntary one may assume that the attitude towards patient evaluations among the participating GPs was predominantly positive. On the other hand, we encouraged the participation of all GPs in a clinic which may have led to the participation of a few more reluctant GPs. We found that the project did not change the à priori attitude of two thirds of the GPs (be it positive or reluctant) while one fifth had become more positive during the process. This leaves us with 14% of the GPs having become less positive which may be a first sign that the project may have raised some barriers to the concept and to the implementation of the results.

The next step towards actual implementation of the results would be if the process stimulated a reflection regarding the patients' perspective on the GPs' practice [19]. 77% answered that they had learnt something from the patient evaluation that they could use in their practice. The results do not tell us whether this was good or bad, but having learnt was in any case a result of a reflective process. The GPs' experienced personal benefit was very high as was the benefit regarding the doctor-patient-relationship and practice' service towards the patients. These significant numbers derive from quite vague questions which nevertheless may have been able to detect an impact of the process and the results that may not have been catched and elaborated in the rather sparse and cursory follow up on the results that was offered within the frame of the DanPEP study.

Around one third of the GPs reported that the awareness within their practice organization to a unique patient perspective on care quality had increased. The complementary result - that the awareness had not increased within two thirds of the practices - contrasts with the above finding of the large proportion of GPs who had learnt from the patient evaluation. In the awareness-questions we asked about the impact on the whole practice staff and hence the contrast may represent a lack of diffusion of the reflections from the GPs and into their organizations (maybe the most significant results were regarding personal qualities). This may be due to a sense of shortness of time for a practice to engage in activities that facilitate the concerted immersion in the matter. It might also have rooted in a reluctance to share the rather personal results with colleagues and staff, which was not supported by our results which indicated an extended willingness to share and discuss the results with others. Throughout the entire process we intended not to point out bad apples but intending to stimulate a process of learning from best practice cases in order to improve practice [20].

Both Davies and Reeves [5,6] state that the impact on the subsequent evaluations of patient evaluation with feedback of results to the organisation and team discussions may not only be due to the implementation of the fed back results but also to the intensified awareness of the staff due to repeted evaluations. This illustrates the difficulty of separating the impacts of different elements of the evaluation-feedback-follow up process.

54% of the GPs reported that they had made changes in their practice following the evaluation and 25% had specific plan to do so (of these 25% there was a fraction which had already made changes but still had plans regarding others topics) and a substantial part of these changes involved the whole organization. So somehow a collaborative effort must have been made. Still, this may have been the result of a top down decision and does not necessarily imply a collaborative reflective process and hence may not have given rise to the change in mindset of the whole staff that should form the base for future improvements in empathy along with communication and information skills and which may only be measurable by applying another patient evaluation.

Most changes and planned changes were of the accessibility to care which was also the group of aspects of practice that were most often criticized by the patients. Adding to the explanation of the large proportion of changes comprised by accessibility aspects may be that in this field the patients and the practices often shared frustrations and hence the motivation for changes was obvious.

Less easy to deal with were the patients' comments on the GPs' empathic and communicative skills. Yet, 21% and 13% of changes and plans regarded the doctor-patient-relationship and information-support matters respectively. Listening to the patient increases the probability that the problem in which you engage during the consultation is actually the problem the patient has. Clinician mindfulness in the consultation increases patient safety [21]. A good doctor-patient-relationship increases the patient's compliance with the treatment [22]. These arguments explain why training empathic and communicative skills does not only serve to improve the patient's experience of care but also increases care quality in the professional perspective [19].

A patient evaluation serves as a tool for improving the patient experienced quality of care and should be conducted in a way that does not affect care quality as viewed in other perspectives (the professional, the organizational and the resource perspective) in a negative way - optimally, there could well be a positive carry over to the other perspectives. We were happy to see that 26% of the GPs experienced an increased job satisfaction following the evaluation. That the job satisfaction of 3.2% of the GPs had deteriorated during the project illustrated clearly why the supervised follow up on the results should - at least regarding some of the GPs - not stop with the end of the sole feedback meeting offered in this study. We know from talking with some of these GPs that they felt left in a limbo. Maybe their job satisfaction was suffering anyway, but by engaging in the project they may have expected to receive better guidance and hence - due to the sparse follow up - were left more frustrated than they were before.

Besides studying the impact of the patient evaluation on the GPs (aim 1) we also set out to identify facilitators and barriers to the implementation of the evaluation results (aim 2). Only half of the GPs had attended the informational meeting prior to the project. Still 83% felt well-informed. This may have been due to the distribution of brief written information. Our analysis did not reveal an association between not being well-informed and not having made changes and plans and hence the informational meeting may not have served as a facilitator for changes - nor did lack of information serve as a barrier. Still, feeling well-informed may have influenced the experience of the process which we did not study further.

We encouraged all GPs in a practice to attend the project and we encouraged the attending GPs to form groups with a view to the later reflection on and working with the evaluation results and 54% of the GPs did so. 88% of the group attendees would choose a group again given the choice. Half of the GPs not attending a group were satisfied with their choice. We found no association between attending a group and the propensity to make changes and plans or a deteriorated job satisfaction, but statistically significantly more GPs who did not attend a group experienced an increased job satisfaction. In their study Wensing and Vingerhoets [14] recommend that the feedback is combined with small group education. We tried that but proved no effect on the outcome (changes and plans). This does not prove against the recommendations but may indicate that the significance of attending a group may show at a later point if the implementations activities are carried on beyond the timeframe of the feedback meeting.

71% of the GPs attended the feedback meeting and 14% of the evaluated GPs found it unpleasant to receive the feedback report containing the results. Not surprisingly, the unpleasantness was associated with results below average but, luckily, not with the propensity to make changes. Hence, as we found also an association between below average results and the propensity to make changes and plans the unpleasantness seems not to have neutralized the motivating effect of the below average results. More scathing was the finding that the unpleasantness was also associated with attending the feedback meeting and that attending the feedback meeting did not facilitate the GPs ability to interpret their results. So even though statistically significantly more GPs who had attended the feedback meeting made changes and plans than those who had not attended, it stands clear that the feedback meetings need a thorough redesign in order to meet the needs of especially vulnerable groups of GPs. In addition, we may have overestimated the impact of the feedback meeting on the propensity to make changes and plans as the GPs who had beforehand chosen not to let the evaluation results affect their practice may have stayed away.

For 267 of the 474 evaluated GPs their feedback reports included the patients' replies to the open-ended questions. These GPs found the results easier to interpret and made statistically significantly more changes and plans. The latter may follow directly of the improved interpretability since some of the patients' comments point directly to clinical processes. The importance of such user centeredness of feedback for facilitating the implementation of the results was pointed out years ago by Tasa et al. in an interview study among all levels of staff in a hospital using patient evaluations for practice improvement [7]. The ultimate user centeredness is represented in the suggestion by Carter et al. to engage patient-practitioner partnership groups in the interpretation of the evaluation results regarding their particular practice [23].

The feedback meetings raised the issue of the lack of a standard for satisfactory patient evaluations. In our information to the GPs we emphasised that an average evaluation score as presented in the feedback reports cannot substitute a lacking standard. The average score is dynamic and changes with the score level of the evaluated GPs. Around half of the GPs will have scores below average - but is that necessarily an evaluation below standard? Still, we found that an evaluation below average made the experience more unpleasant for the GP, made the GP less positive towards patient evaluations, decreased job satisfaction and made the GP less prone to recommending an evaluation to a colleague. So we have to find a way to deal with the GPs' demand for a benchmark still taking into account that we don't have - and probably never will be able to set - an outcome standard for patient evaluations. One may state that the GPs should strive for 100% positive evaluations as the final indication of true individualized patient-centred care but this may not be realistic in real everyday practice and not desirable from a cost benefit point of view as it may be too costly measured in the economic and organizational perspective and compromise the professional perspective on care quality.

### Strengths and limitations

This study had a high number of participating GPs and a high response rate to the survey among the evaluated GPs, which yielded high precision and minimised selection bias.

The choice to use the EUROPEP questionnaire was made at an early stage of the DanPEP study and not entirely in order to meet the aims of the present study. Addressing the aspects of clinical practice which have been shown to be of highest relevance to the patients' experience of quality in general practice care [1,2] and being validated in an international [16,17] as well as a national setting [24,25] it served as a sound tool for meeting the quality claims of the participating GPs. We assumed that addressing aspects of practice that were important for the patients' experience of care quality would enhance the impact of the evaluations. Realising that quantitative data may be difficult to interpret in terms of daily practice we chose to add the open-ended questions in order to facilitate this process. The different methods for distribution of the patient surveys and the different use of reminders had a slight effect on the evaluation results [18,26] but as this effect was the same throughout an entire county and as we did not present results including more counties this did not affect the ranking of the individual GP above or below the county average.

In this study there is a risk of multi significance due to testing several differences between groups. With the Bonferroni criterion [27] a significance level of 0.004 (=0,05/12) should be used to be sure that we do not make type-1-errors. Further, we included only the variables relevant to test the hypotheses made beforehand and thus did not test for all differences regarding all available data. The main purpose of obtaining patients' evaluations was to motivate GPs to introduce changes that would improve the patient-experienced quality of care. To measure the effect of the evaluations, the feedback and the subsequent quality-improving activities, another patient evaluation would therefore, ideally, have to be conducted at a later point of time, and the results compared with those of a control group who had received no feedback of their results. This was done in the study by Vingerhoets showing no effect of feeding back on the evaluation results [15]. Time constraints did not allow such a design in our study. In addition, the results of such a comparison would, however, be difficult to interpret as a change in patient evaluation could reflect both changes in the patients' frames of reference and the actual results of improvement of care [28]. As future changes in patient evaluations will derive from immediate changes in GPs' attitudes and practices, we therefore chose to use those as surrogate outcome measures.

At the outset of this study we were aware that including GPs by invitation was likely to provide us with an à priori forthcoming study population which probably would result in more beneficial evaluation results than if the study had engaged all GPs. Nevertheless, analyses conducted within the framing DanPEP-study showed that the county average evaluation did not increase with the increasing fraction of participating GPs in the included counties. We realised what was also suggested by Wensing and Vingerhoets [14] that praising evaluations may not be as stimulating for a GP as more critical ones and that offering patient evaluation to volunteering GPs may not provide encouragement enough to participate in the project to those GPs who would be the most likely to gain. These facts may have made the study population (the evaluated GPs) too homogenous for our purpose.

Even though the information and the feedback procedures were the same throughout the two-and-a-half-year study period, the GPs' attitude to patient evaluations may have changed (which is supported by the increasing attendance as the DanPEP study proceeded) and we may have improved our procedures in light of the experience gained. Furthermore, follow-up activities vis-à-vis the evaluations were carried out to different extents in different counties.

As the questionnaire was received by the GPs three to 17 months after the patient evaluation, their replies may have suffered recall bias to different extents. Still, we found no correlation between the time elapsed since the patient evaluation and the GPs' having learnt from the evaluations and having made changes and plans. We found that GPs attending a feedback meeting made more changes and plans than those who did not, but this may be ascribed to selection bias and does not necessarily reflect a benefit from the meetings.

Finally, evaluation of the effect of a quality improvement project by the project group itself is bound to raise the question whether this has biased the results. Ideally, such an evaluation should be carried out by a third part. From the outset of the DanPEP study this evaluation was agreed on with the stakeholders. Throughout the project there was an open and frank dialogue between the GPs and the project group that left no impression of a pleasingly positive attitude towards the project.

## Conclusions

In a set up with voluntary informed participation in a patient evaluation survey using a validated questionnaire, individual written feedback with quantitative as well as qualitative data and a single feedback meeting with group discussions of the results we found a significant impact on the GPs regarding satisfaction with the process and attitude towards patient evaluations, GPs' attention to the patients' perspective on care quality and their job satisfaction which was for one in four improved but for a smaller share had deteriorated. Three in four GPs felt that they had learnt from the evaluation and around 60% had made or planned changes in their practice of assumed benefit to the quality of care. Yet, the impact on the GPs contrasted with the only one in three GPs who reported a raised attention to the patients' perspective on care quality in their entire practice (doctors as well as staff).

Our study pointed out a few facilitators for implementation of the results. Adding qualitative data to the quantitative results showed to improve the interpretability of the fed back results via-à-vis daily practice and hence increase the propensity to make changes. The significant willingness (maybe even a need) to share and discuss the results demonstrates an openness to a group based implementation process.

The barriers pointed out in this study related to the feedback reports and to the feedback meetings and the implementation process. Being benchmarked against the average seemed to have paralysed some of the GPs with below average results thus preventing them from benefitting from the feedback meeting. In addition this experience seemed to have affected the attitude to patient evaluations negatively. Many GPs found the reports difficult to interpret which was partly overcome by adding the qualitative results. Still the layout may have constituted a hindrance to getting full benefit of the evaluation.

Leaving the evaluated GPs to their own initiative after the feedback meeting may have left some loose ends which were never followed up - not necessarily due to a lack of interest but maybe merely lack a room for reflection. Hence the lack of follow up may have prevented the full benefit regarding attention and attitude together with improving activities and further it may have prevented the GPs who needed it from having the opportunity to change frustration and job dissatisfaction into personal and professional development.

### Implications

Following the present study we recommend that following future patient evaluations the feedback to the GPs should contain both quantitative and qualitative results and that it is presented in a way that ascertains its interpretability. Searching for the ideal way to present the evaluation results with an illustrative benchmark that makes sense without being able to provide a relevant standard for the outcome of a patient evaluation will probably also in the future be an ongoing struggle. We still recommend a plenary meeting with comprehensible guidance for the interpretation of the results. It may be sensible to provide the GPs with their results prior to the meeting that initiates the implementation process thus offering a chance to reflect in private. Still, attention should be raised to those in risk of skipping a needed guidance toward improvement and who, in the present set up, are indeed in risk of being left in limbo.

Furthermore, to ensure that the GPs derive maximum benefit from the patient evaluation, they should be offered structured, differentiated and supervised activities over a period of time to follow up on the results relating to particular aspects of the evaluation. Such activities may very well take place in of groups of GPs who would thus benefit from fruitful interaction between colleagues. In our study the GPs expressed a benefit from participating as part of a group which was not reflected in the outcome of the project. We have reason to believe that the benefit of the groups will show when the process is followed up as described in the above.

As many of the evaluated aspects relate not only to the GPs but indeed also to the practice and staff it is crucial that the impact that the evaluations were shown to have on the GPs is carried through to the entire organisation in order to raise the attention and improve the skills of all the involved professionals. This raises yet another issue if patient evaluations should continue only to be offered to the GPs in a general practice organisation where nurses and other staff members are increasingly engaged in unassisted (yet supervised) consultations.

This study was carried out in a Danish general practice setting. Even though general practice is organised differently in other countries we consider our results and subsequent recommendations widely generalisable to any general practice setting in which patient evaluations are carried out in a quality improvement perspective. Some of the recommendations may be also carried through to a hospital setining.

## Abbreviations

GP: general practitioner; CEP: Chronically ill patients Evaluate general Practice; IPQ: Improving Practice Questionnaire; EUROPEP: The European Patients Evaluate General/Family Practice; DanPEP: Danish Patients Evaluate Practice.

## Competing interests

The authors declare that they have no competing interests.

## Authors' contributions

HH conducted the patient evaluation study. HH, PV and FO elaborated the questionnaire to the evaluated GPs and the study design. HH conducted the following survey. HH made the data analyses. All authors contributed to, read and approved of the final manuscript.

## Pre-publication history

The pre-publication history for this paper can be accessed here:

http://www.biomedcentral.com/1471-2296/12/116/prepub

## Supplementary Material

Additional file 1 **Danish general practice**. A description of the organisation of general practice in the Danish health care organisation. Click here for file

Additional file 2 **Changes in practice**. A full tabulation of all changes reported by the responding GPs made following the patient evaluation of their practice. Click here for file
