# Supplementary material for: General practitioners' experience and benefits from patient evaluations
Source: BMC Fam Pract. 2011 Oct 31;12:116. doi: 10.1186/1471-2296-12-116 (PMC3217866; doi:10.1186/1471-2296-12-116)
Supplement: Additional file 1 — Danish general practice. A description of the organisation of general practice in the Danish health care organisation. [file 1471-2296-12-116-S1.DOC]

**Danish general practice**

Denmark (5.5 mio. inhabitants) was at the time of the study divided into 16 health administrative regions (now 5) with 50,000 to 625,000 inhabitants. In each region the Public Health Insurance controls the number of general practitioners. 98% of the citizens are listed with a local GP and receive tax-supported free medical care. The GPs are self-employed, but are reimbursed by the Public Health Insurance both per capita (citizens on the GP’s list) and on a fee for service basis (30%/70%). The GPs serve as gatekeepers vis-à-vis the rest of the health care system (i.e. access to a hospital or specialist care implies referral by a GP) and are responsible for the care of all registered patients 24 hours a day. The GPs in a region co-operate about the out-of-hours care. The GPs are the head and manager of their own practice either alone in single-handed practice, in cooperatives of single-handed practices or in partnership practice in which case the GPs share the management. 65% of the GPs work in partnership practices. The GPs employ their own staff and are fully responsible for the economy of the clinic. It is still uncommon for GPs to employ a separate practice manager. Hence, the GPs in the DanPEP study were approached both in their role as GPs and as practice managers.
