# Supplementary material for: General practitioners' experience and benefits from patient evaluations
Source: BMC Fam Pract. 2011 Oct 31;12:116. doi: 10.1186/1471-2296-12-116 (PMC3217866; doi:10.1186/1471-2296-12-116)
Supplement: Additional file 2 — Changes in practice. A full tabulation of all changes reported by the responding GPs made following the patient evaluation of their practice. [file 1471-2296-12-116-S2.DOCX]

**Additional File 2. Changes in practice**

Of the 474 responding GPs 264 (56%) had taken the opportunity to describe the changes (mostly more than one) that they had made in their practice following the patient evaluation.

| **The GP-patient relation** | | | | | | | | | | | | | | | | |
| --- | --- | --- | --- | --- | --- | --- | --- | --- | --- | --- | --- | --- | --- | --- | --- | --- |
| Improved communication 4 | | | | | | | | | | | | | | | | |
| Raised awareness of the consultation process 5 | | | | | | | | | | | | | | | | |
|  | I listen more carefully to the patient 16 | | | | | |  | |  | |  | |  | |  | |
|  | Become more present minded 12 | | | | | |  | |  | |  | |  | |  | |
|  | Seem less busy or stressfull 7 | | | | | |  | |  | |  | |  | |  | |
|  | More conscious use of body language 2 | | | | | | | |  | |  | |  | |  | |
|  | Seem more warm and emotional 1 | | | | | |  | |  | |  | |  | |  | |
|  | Involve the patient more in decisions 1 | | | | | |  | |  | |  | |  | |  | |
|  | Become more structured and in control of the consultation 5 | | | | | | | | | |  | |  | |  | |
|  | Become better at keeping the time 19 | | | | | |  | |  | |  | |  | |  | |
|  | Took a training course in communication skills 2 | | | | | | | |  | |  | |  | |  | |
|  | Is having personal supervision 2 | | | | | |  | |  | |  | |  | |  | |
| More emphasis to the professional/medical quality 4 | | | | | | |  | |  | |  | |  | |  | |
|  | Took courses in specific medical competencies 3 | | | | | | | |  | |  | |  | |  | |
| Read the filed notes from the latest contacts with patient prior to the consultation 8 | | | | | | | | | | | | |  | |  | |
| Better information to the patients 14 | | | | | | | | | | | | | | | | |
|  | Better information about examinations and treatment 7 | | | | | | | | | |  | |  | |  | |
|  | Better information about I relation to referral 3 | | | | | | | |  | |  | |  | |  | |
|  | Become more attentive to the patient’s understanding and acceptance of the conclusion to the consultation 3 | | | | | | | | | | | | | | | |
|  | Documentation of given information in the file 1 | | | | | | | |  | |  | |  | |  | |
|  | | | | | | | | | | | | | | | | |
| **The practice-patient relation** | | | | | | | | | | | | | | | | |
| Better information to patients about pratice’ structure and routines 15 | | | | | | | | | | | | | | | | |
| Better information about waiting time 2 | | | | | | |  | |  | |  | |  | |  | |
| Better communication between practice and patients 7 | | | | | | | | |  | |  | |  | |  | |
| Improving the routine for communicating test-results to the patients 1 | | | | | | | | | | |  | |  | |  | |
|  | | | | | | |  | |  | |  | |  | |  | |
| **Internal organisation** | | | | | | |  | |  | |  | |  | |  | |
| Changed working schedules and redistributed tasks and competencies to and between staff 19 | | | | | | | | | | | | | | |  | |
| Trained staff in the consultation process 5 | | | | | | |  | |  | |  | |  | |  | |
| Improved coutesy and kindness in reception and telephone 10 | | | | | | | | | | | | | | | | |
| Improved discretion in reception and waiting room 11 | | | | | | |  | |  | |  | |  | |  | |
| Optimised internal conference and sharing of information about patients 5 | | | | | | | | | | |  | |  | |  | |
| Improved the quality of prescriptions 4 | | | | | | |  | |  | |  | |  | |  | |
|  |  | |  |  |  | |  | |  | |  | |  | |  | |
| **Accessibility** | | | | | | | | | | | | | | | | |
| Improved accessibility 18 | | | | | | | | | | | | | | | | |
|  | Changed working schedule/time planner 17 | | | | | | | | | | | | | | | |
|  | Introduced ”consultation without appointment” for a limited period every day 7 | | | | | | | | | | | | | | | |
|  | *Reduced the ”consultation without appointment” 1* | | | | | | | |  | |  | |  | |  | |
|  | Introduced scheduled time to see patients with urgent problems without delay 8 | | | | | | | | | | | | | |  | |
|  | Extended the scheduled time for certain appointments 4 | | | | | | | | | | | | | | | |
|  | *Shortened the scheduled time for certain appointments 4* | | | | | | | | | |  | |  | |  | |
| Reduced my personal accessibillity 5 | | | | | | |  | |  | |  | |  | |  | |
| Closed the patient list 1 | | | | | | |  | |  | |  | |  | |  | |
| Improved accessibillity by telephone 22 | | | | | | | | | | | | | | | | |
|  | Extended the telephone opening hours 7 | | | | | |  | |  | |  | |  | |  | |
|  | More persons to answer the telephone 4 | | | | | |  | |  | |  | |  | |  | |
|  | Improved preadmission evaluation 7 | | | | | |  | |  | |  | |  | |  | |
|  | Bought and introduced a new telephone system 39 | | | | | | | |  | |  | |  | |  | |
| Initiated a project on accessibillity 8 | | | | | | |  | |  | |  | |  | |  | |
| Introduced a new/another electronic file- and patient-management system 3 | | | | | | | | | | | | |  | |  | |
| Introduced internet access 1 | | | | | | |  | |  | |  | |  | |  | |
|  | Introduced a homepage for the clinic 7 | | | | | |  | |  | |  | |  | |  | |
|  |  | | Introduced electronic booking 14 * | | | | | |  | |  | |  | |  | |
|  |  | | Introduced electronic renewal of prescriptions 12 * | | | | | | | |  | |  | |  | |
|  |  | | Introduced e-mail consultation 35 * | | | | | |  | |  | |  | |  | |
|  | | | | | | | | | | | | | | | | |
| **Changes in staff** | | | | | | | | | | | | | | | | |
| Employed extra staff 9 | | | | | |  | |  | |  | |  | |  | |  |
| Changed staff 3 | | | | | |  | |  | |  | |  | |  | |  |
| Dismissed staff 1 | | | | | |  | |  | |  | |  | |  | |  |
| Engaged an extra GP-trainee 1 | | | | | |  | |  | |  | |  | |  | |  |
| Entered a partnership practice 1 ** | | | | | |  | |  | |  | |  | |  | |  |
| Took in an extra GP-partner 1 ** | | | | | |  | |  | |  | |  | |  | |  |
|  | | | | | | | | | | | | | | | | |
| **Changes of premises** | | | | | |  | |  | |  | |  | |  | |  |
| Built a new clinic 2 ** | | | | | |  | |  | |  | |  | |  | |  |
| Moved to new premises 3 | | | | | |  | |  | |  | |  | |  | |  |
| Reconstruction/redecoration 5 | | | | | |  | |  | |  | |  | |  | |  |
| Renovating the waiting room 8 | | | | | |  | |  | |  | |  | |  | |  |
|  | | Installed a computer screen in the waiting room for information purposes 1 | | | | | | | | | | | | | |  |
|  | | Introduced soft music in the waiting room 3 | | | | | |  | |  | |  | |  | |  |
|  | | Placed an fish tank in the waiting room 1 | | | | | |  | |  | |  | |  | |  |

* Mandatory from the 1^st^ of January 2009 as part of the GP contract with the Health Insurance

** Process accelerated but not entirely prompted by the patient evaluation
